# Supplementary material for: Enhanced quantum signature scheme using quantum amplitude amplification operators
Source: PLoS One. 2021 Oct 8;16(10):e0258091. doi: 10.1371/journal.pone.0258091 (PMC8500455; doi:10.1371/journal.pone.0258091)
Supplement: Attachment — Submitted filename: Response to reviewers.docx [file pone.0258091.s001.docx]

Reviewer #1: The paper describes an extension towards the quantum signature scheme based on Grover's quantum search algorithm related diffusion / partial diffusion / amplitude amplification operators along the lines of the publication of A. Younes "Enhancing the security of quantum communication by hiding the message in a superposition" from 2011 (<https://doi.org/10.1016/j.ins.2010.09.017>). The current submission however does not offer a sufficient conceptual advancement from the mentioned paper to justify its publication in PLOS ONE. The main concept of the paper has been described previously in its major part, and thus both the core of the proposed QDS along with its security analysis have been to a high extent repeated after the previous result largely unchanged on a conceptual level.

The paper does not provide sufficient disclosement of the fact that in terms of the protocol's conceptual layer it is mainly repeating the idea of a previous paper of one of the co-authors from 2011. Hence in its current formulation the proposed protocol lacks the novelty justifying its PLOS ONE publication. If the paper is to be reconsidered for the PLOS ONE publication, a significant (major) revision would be required, first in-detail addressing the issue of basing the proposal on the 2011 paper (including indications of which parts of the protocol are essentially repeated) and secondly clearly explaining the conceptual progress from this previous paper that could justify significant novelty required for the publication.

*Author response: Thank you very much for the comment. The paper has been reorganized by adding section 2 to separate the work done in ( <https://doi.org/10.1016/j.ins.2010.09.017>) from the contribution of the paper.

Furthermore the security analysis of the protocol in its current formulation is not very convincing and should be extended to justify the claims made on the security of the protocol. The soundness of the concept should also be elaborated on.

*Author response: Thank you very much for the comment. The security analysis of the protocol has been discussed and explained in page 11, lines from 222 to 236.

Furthermore issue of quantum memory requirement for the proposed QDS should be discussed in view of the recent progress with memory-less QDS.

*Author response: Thank you very much for the comment. The issue of quantum memory requirement is discussed and explained in page 12 and 13, lines from 259 to 271.

------------------------------------------------------------------------

Reviewer #2:


1. Line 78, there should be a 3rd qubit there in state |1>

Author response: Thank you very much for the comment.

2. I assume the message to be signed is a S-qubit quantum state (if yes, can this be arbitrary or only a product state?). Can the authors clarify that.
E.g., on line 100, "Alice sends the message to the receiver on a public channel" -> "... public quantum channel"

Author response: Thank you very much for your valuable comment that enables us to enhance and clarify the paper. The announcement of the message is explained in page 6, lines 115 and 116.

3. On line 111, what do the authors mean by "QKD protocol with size 3S qubits"? Is 3S the length of the established key? In that case, the QKD requires significantly more than 3S qubits to distill a 3S-length key.

Author response: Thank you very much for your valuable comment that enables us to clarify the paper. The requirement of the QKD protocol is discussed and explained in page 6, lines from 117 to 120.

4. Regarding the QKD, it is not clear from the text that it has to go through the TC (as shown in the Fig. 1). Can Alice and Bob perform the QKD without the TC?

Author response: Thank you very much for the comment. In the scheme, TC sends a secret key to Alice to sign the message (page 6, line 117) then TC verifies the signature using the shared key, then TC sends the signed message to Bob based on a shared secret key that Bob sends to TC (page 9, line 173). If Alice and Bob perform the QKD without TC, TC cannot verify the signed message that Alice sends because TC does not have a secret key and cannot sign the message again to send it to Bob.

5. Sign is wrong on line 87, the minus should appear on the other 3 states

Author response: Thank you very much for the comment. We would like to inform you that the equation is correct, and the minus sign is on state |00> only because Grover’s operator is applied on state |00> (no perfect superposition and no oracle for marking).

6. During the verification scheme, the authors mention that the public message
(I assume string of received qubits) is "compared" against the decoded one
received via the channel. This implies the receiver must have access to a
long-lived quantum memory, which should be included in the resource analysis
of Table 2.

Author response: Thank you very much for your valuable comment that enables us to clarify the paper. The announcement of the message is explained in page 6, line 115 and 116. The issue of quantum memory requirement is discussed and explained in page 12 and 13, lines from 259 to 271. The need of the long-lived quantum memory is included in the resource analysis
of Table 2.

7. Can the authors comment more on the role of the TC and the attack model?
What exactly can Eve intercept/modify?

Author response: Thank you very much for the comment. The security analysis of the protocol has been discussed and explained in page 11, lines from 222 to 236.

8. The encoding procedure during the signing phase is not very clear. I urge the authors to write the encoding as a set of 3 quantum circuits, one for each case, which will make the procedure more clear for the reader.

Author response: Thank you very much for your valuable comment that enables us to clarify the paper. The cases of the signing phase are explained and discussed in page 8, lines from 150 to 167.
