# Supplementary material for: Enhanced quantum signature scheme using quantum amplitude amplification operators
Source: PLoS One. 2021 Oct 8;16(10):e0258091. doi: 10.1371/journal.pone.0258091 (PMC8500455; doi:10.1371/journal.pone.0258091)
Supplement: Attachment — Submitted filename: Response to reviewers.docx [file pone.0258091.s002.docx]

Reviewer #2: I appreciate that the authors addressed some of my concerns, and clarify some
of the material in the paper.
My only worry is that the current paper is not innovative enough in comparison with Ref. 12. I appreciate that the authors introduced Sec. 2, in which they succinctly describe Ref. 12, however I find the progress made in the current manuscript to be relatively incremental compared to Ref. 12, and therefore does not meet the PONE standards of novelty.

*Author response: Thank you very much for the comment. The paper has been reorganized by adding section 5 to discuss the innovation done in our work compared with Ref. 12.
